# IRT scoring procedures for TIMSS data

**DOI:** 10.1016/j.mex.2019.06.015

**Published:** 2019-06-21

**Authors:** Gregory Camilli, John A. Dossey

**Affiliations:** a Rutgers University, United States; b Illinois State University, United States

**Keywords:** Empirical subscore estimation, Multidimensional scoring, Item response theory, IRT, Exploratory factor analysis, Mathematics achievement, Empirical subscores, International assessment, Diagnostic information, TIMSS

## Abstract

In large-scale international assessment programs, results for mathematics proficiency are typically reported for jurisdictions such as provinces or countries. An overall score is provided along with subscores based on content subdomains defined in the test specifications. In this paper, an alternative method for obtaining *empirical subscores* is described, where the empirical subscores are based on an exploratory item response theory (IRT) factor solution. This alternative scoring is intended to augment rather than to replace traditional scoring procedures. The IRT scoring method is applied to the mathematics achievement data from the Trends in International Mathematics and Science Study (TIMSS). A brief overview of the method is given, and additional material is given for validation of the empirical subscores. The ultimate goal of scoring is to provide diagnostic feedback in the form of naturally occurring item clustering. This provides useful information in addition to traditional subscores based on test specifications. As shown by Camilli and Dossey (2019), the achievement ranks of countries may change depending on which empirical subscore of mathematics is considered. Traditional subscores are highly correlated and tend to provide similar rank orders.

• The methods takes advantage of the TIMSS sampling design, specifically pairs of jackknife zones, to aggregate categorical to higher-order sampling units for IRT factor analysis.
• Once factor scores are estimated for sampling units and interpreted, they are aggregated to the jurisdiction level (countries, states, provinces) using sampling weights. The procedure for obtaining standard errors of jurisdictional level scores combines cross-sampling-unit variance and Monte Carlo sampling variation.
• Full technical details of the IRT factoring procedures are given in Camilli and Fox (2015). Fox (2010) provides additional background for Bayesian item response modeling techniques. The estimation algorithm is based on stochastic approximation expectation-maximization (SAEM).

## Graphical abstract

Sampling units are pairs of TIMSS jackknife zones. Aggregated θ and its corresponding SE are estimated with sampling weights.

**Figure:**
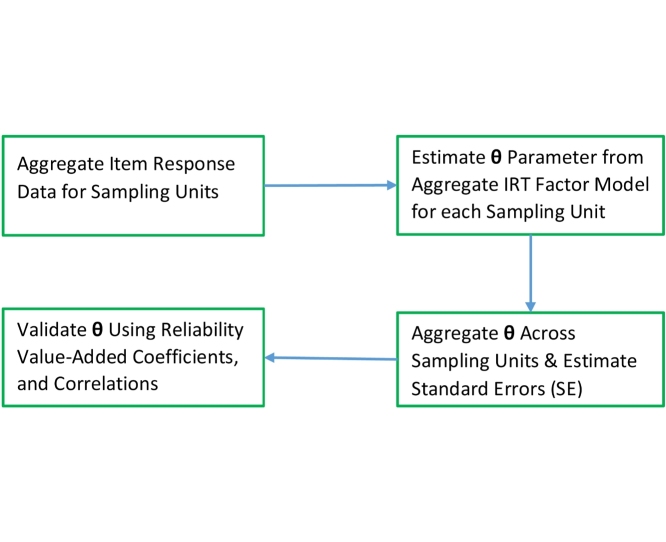

**Specifications Table**

**Table:** 

Subject area:	Mathematics
More specific subject area:	Estimating multidimensional subscores for international comparisons in mathematics achievement
Method name:	Empirical subscore estimation
Name and reference of original method:	Camilli, G. & Dossey, J. A. (2019). Obtaining multidimensional national profiles for TIMSS 2007 and 2011 mathematics. Journal of Mathematical Behavior. https://doi.org/10.1016/j.jmathb.2019.02.001. Camilli, G. & Fox, J.-P. (2015). An aggregate IRT procedure for exploratory factor analysis. Journal of Educational and Behavioral Statistics, 40, 377-401. https://doi.org/10.3102/1076998615589185.
Resource availability:	Gregory Camilli

## Methods details

### Introduction

In large-scale international assessments, results for mathematics achievement are typically reported for jurisdictions such as provinces or countries. An overall score and content subscore scores are provided, but the most conspicuous feature of reporting is a set of ranks based on total scores. In this paper, a method for interpreting factor structures and deriving associated empirical factor scores is described relative to data from the Trends in International Mathematics and Science Study (TIMSS. A brief overview of the method is given below, and additional details for subscore estimation are given in Camilli and Fox [1]. Fox [2] provides a thorough background for Bayesian item response modeling techniques. In the companion paper [3] to the present article, a discussion of the TIMSS framework for mathematics assessment is provided and substantive results are broken down by participating countries.

### Traditional and empirical subscores

A single score on an assessment and its associated rank encourage policy makers to view student achievement in terms of a “horserace” among countries or states. Describing performance in terms of subscores relating to different kinds of mathematics skills would at first appear to encourage more strategic use of test information. For this purpose, framework-based subscores for TIMSS can be obtained and examined with International Data Explorer made available by the National Center for Education Statistics at https://nces.ed.gov/surveys/international/ide/. Alternatively, subscores can be devised empirically using exploratory factor analysis.

### Data

Data were obtained from the TIMSS 2007 mathematics for Grade 4 [4]. In TIMSS 2007, there were 59 jurisdictions with about 150 schools per jurisdiction and over 200 items matrix sampled over students (who each typically take about 25 items). It is convenient to refer to the higher-order units as *countries*, but TIMSS participants may include jurisdictions such as countries, states, or provinces within a country. With this usage in mind, the number of countries differs by both grade and topic (science or mathematics). For Grade 4, for example, there are 41 jurisdictions, 177 items, and 194,000 students. Students receive a subset of items (matrix sampling), but each item is paired with all other items within a country so that items can be analyzed simultaneously. Matrix sampling is a common design for large-sale assessments that do not report scores at the student level. The method described in this paper and Camilli and Fox [1] works well for TIMSS data, but has not yet been extended to other related methods of sampling.

A large number of items were not presented to individuals at the school level due to item matrix sampling procedures. Thus, school is not a practical unit for analysis. To address this sparseness issue, *jackknife zones* (JKZ) were used as units, where JKZs are formed by sorting schools on both explicit and implicit background variables and then pairing adjacent schools [5]. This process can be thought of as creating pairs of schools. This resulted in approximately 3000 level 2 units (or pairs) at each year-grade combination. The data set for analysis then consisted of a set of three variables for each jackknife unit within a country for each item: the total sample size for the given item, the weighted number of correct responses, and the weighted number of incorrect responses. Because individuals’ responses to an item within a sampling unit (jackknife zone) are aggregated, this can substantially reduce both the size of the data set and processing time. This modeling approach is a natural extension of the work by Mislevy [6,7] and Mislevy and Bock [8].

### Aggregate factor analysis

The data were then analyzed with the aggregate model of Camilli and Fox [1]. The goal of exploratory factor analysis at the higher-order level is to discover clusters of items that perform similarly across countries. The clusters are identified by expert interpretation of a loading matrix resulting from an exploratory factor analysis, and then scores are derived that correspond the factor structure. In short, the clusters are the “factors” of factor analysis, and the empirical subscores are factor scores.

### Factor selection and interpretation

A three-factor solution was obtained at fourth grade for TIMSS 2007 and 2011 after transforming the initial solution to simple structure using the oblimin rotation. In both grades, the factor structure was observed to be nearly the same for 2007 and 2011. Choice of the number of factors was driven by three factors. First, eigenvalues are a byproduct of the aggregate model method, and these serve as a rough guide when employed in scree plots. This procedure usually indicated a factor structure of 2–5 factors. For example, from Table 4 of Camilli and Fox [1], the first five eigenvalues are given for Grade 4 TIMSS data in 2007. In order these are 147.14, 10.89, 7.83, 2.88, and 2.63. The scree plot indicated a break between the 3- and 4-factor solutions, though factors 4 and 5 showed moderate stability between 2007 and 2011. Next, substantive interpretations of the factors loadings were developed. Only factors with clear substantive interpretations were considered. Third, we conducted reliability analyses (reported below), and choose factors with cross-cycle (2007, 2011) correlations of .89 or higher. Finally, factor scores **θ***_ik_* for each country *i* for each *JKZ k* on each rotated factor were generated.

### Factor interpretation

There are many items in TIMSS, and an exploratory analysis yields m*Q factor loadings for *m* items and *Q* factors. For this reason, a method was constructed for interpreting factors based on two different statistics. First, the 10 highest loadings were considered in developing factor descriptions, which were determined by examining both the actual items and item information provided on the TIMSS website. Second, to describe factor resolution the full range of loadings were examined to determine whether the majority were positive for any given factor. As an illustration, consider TIMSS 2011 at grade 4 for 178 total items. The following tallies were used to evaluate the factor structure: the number of positive loadings, the number of loadings with magnitude <.1, and the number of negative loadings <−.1. For factor 1, these tallies were 176, 4, and 1. One moderately-sized negative loading was observed (this item involved fractions and decimals), with 75 positive loadings higher in magnitude. For factor 2, the tallies were 130, 77, and 18. One moderate negative loading was observed, while 20 positive loadings higher in magnitude were observed. For factor 3, the tallies were 127, 80, and 13. One moderate negative loading was observed, with 15 positive loadings higher in magnitude. Similar results were obtained for other year/grade combinations.

Choosing the largest negative loading to evaluate the number of larger positive loadings tends to understate factor resolution. If the second highest negative loading was chosen, the number of positive higher loadings higher in magnitude roughly doubles for factors 2 and 3. We also used this procedure to confirm the number of factors chosen; factors not chosen (factors 4 and 5) performed worse on these tally statistics. Note also, that items used to interpret factors always had substantially higher loadings than the highest negative loading. A substantive interpretation of each factor is provided below.

For 2007 Grade 4 TIMSS data, items not sharing the fundamental theme suggested by the factor name tended not to occur for factors 2 and 3, with the caveat that as the loadings approached zero (all items have loadings because this is EFA), there was less consistency with the overall theme. For factor 1 (having to do with visual aspects of problem-solving) some items did at first appear inconsistent. One such item concerned whole numbers: “Maria has 6 red boxes. Each red box has 4 pencils inside. She also has 3 blue boxes. Each blue box has 4 pencils inside. How many pencils does Maria have altogether?” Another whole number question asked “How much do the apples weigh in grams? This item included a drawing of a scale with apples on the weight tray and an analog read out. Thus, both items upon further examination were determined to reflect the overall factor theme due to their strong visual elements. The factors are described in more detail below. More information on factor interpretation can be obtained by contacting the first author, including the TIMSS items with the highest loadings of the three mathematics factors for 2007 Grade 4.

### Factor Descriptions Grades 4 and 8

Below, the factors are briefly described. Full interpretations as well as sample items loading on the factors are given in Camilli and Dossey [3].

• *Factor 1_4*. Factor 1 involves items requiring numerical reasoning with both charts and symbols, graphical reasoning related to data display, interpreting geometric shapes, and locating objects on a map. This factor can be thought of as reasoning with visual scaffolds, and is shortened to “Graphical Reasoning.”
• *Factor 2_4*. Factor 2 involves whole numbers and number sentences. These items all involve solving for an unknown rather than simple counting. This subscore is labeled “Solving for Unknowns.”
• *Factor 3_4*. Factor 3 is clearly focused on fractions and decimals from a variety of perspectives. This factor is labeled “Fractions and Decimals.”
• *Factor 1_8*. Factor 1 predominantly involves items requiring applications of numerical skills in contextual settings. This factor is labeled simply as “General Numerical.”
• *Factor 2_8*. Factor 2 involves solving mathematical problems, but here, the focus is clearly on algebraic reasoning involving rates, ratios, and proportions. For shortened reference, this factor is labeled “Algebra and Ratios.”

### Computing country level factor scores and standard errors

The aggregate MIRT (multidimensional IRT) model was proposed by Camilli and Fox [1] for addressing computational issues with large numbers of items and groups. They showed how factor scores $\theta_{ik}$ could be generated for jackknife zone *k* of country *i* within Gibbs estimation cycles. Note we refer to θ as a vector (of length *Q*) of empirical subscores. The new methodology reported here is how this information is aggregated to the country level in three steps:

• Compute the aggregate sampling weights of each i,k unit by $w_{ik}=\sum_{j}w_{ijk}$, where i, j, and k subscript countries, individual examinees within country, and sampling zones within countries, respectively.
• Compute the average country level score by ${\bar{\theta }}_{i}=\sum_{k}w_{ik}\theta_{ik}/\sum_{k}w_{ik}$ aggregating across sampling zones. The vector θ holds q=1,2,...,Q empirical subscores obtained from the aggregate factor analysis.
• Approximate the standard error (SE) for each individual subscore $\theta_{qi}$ within country *i* by
  ○ $sb_{qi}^{2}={\sum_{k}w_{ik}\left(\theta_{qik}-{\bar{\theta }}_{qi}\right)}^{2}/\sum_{k}w_{ik}.$ This is the weighted between sampling zone variance.
  ○ $sw_{qi}^{2}=\sum_{k}w^{ik}s_{qik}^{2}/\sum_{k}w_{ik},$ where $sw_{qi}^{2}$ is the estimate of MCMC error associated with the empirical subscore obtained through Gibbs sampling.
  ○ Given $n_{i}$ of sampling zones within country *i* (about 75), approximate the standard error as: $SE\left(\theta_{qi}\right)={\left(\left(sb_{qi}^{2}+sw_{qi}^{2}\right)/n_{i}\right)}^{1/2}$.

For TIMSS official score reports, the SEs of country means is obtained by a jackknife replication procedure. For the present analysis, SEs were approximated by a simpler method. However, the correlation between the approximate SEs for the first factor and the jackknife SEs for the overall TIMSS score were *r* = .85 and *r* = .75 at Grades 4 and with outliers (Dubai and Qatar) removed.

### Reliability of empirical subscores

Obtaining parallel forms reliability is problematic in the case of TIMSS, because there are not parallel forms in the classical sense, but rather multiple spiraled forms containing matrix-sampled items. In reports of testing outcomes, reliability coefficients for TIMSS scores are not given (e.g., [9]). However, to understand the reliability of the empirical subscores, two approaches were taken: simulation and test-retest. The simulation approach consisted of four steps: (1) estimate item parameters and level 2 scores with the aggregate model and designate these as *generating parameters*, (2) simulate individual-level item responses based on generating values, (3) carry out a new analysis of the simulated data, and (4) correlate generating level 2 scores (*θ*) with those estimated from the simulated data. This procedure was carried out by Camilli and Fox [1]. They found correlations of .95, .90, and .89 for the first three factors in 2007 Grade 4 Data. On the other hand, correlations for the fourth and fifth factors were notable lower at .54 and .43. As noted below, the decision for choosing the number of factors involved this reliability as one of three criteria.

Because item response data were generated according with an individual MIRT model that is consistent with the MIRT model used for analysis, these reliabilities may be inflated. Actual data may vary from MIRT assumptions. For an alternative to the simulation approach, a test-retest reliability was carried out for the three 2007 and 2011 subscores at Grade 4. Cross-year correlations of .96, .95, and .89 were found for country-level empirical subscores. Similar results were found at Grade 8 for 2007 and 2011 with reliabilities for the first two factors at .96 and .93 (there was no indication of additional factors at Grade 8). These latter coefficients may be deflated due to national changes in instruction or curriculum across the four-year interval.

### Subscore value-added

As shown in Table 1, the intercorrelations among empirical subscores in $\theta_{i}$ were low to moderate (.15–58), and higher in Grade 8 than in Grade 4. Thus, factor-based subscores provide more distinct information for understanding national-level achievement. In contrast, intercorrelations among TIMSS domain scores are much higher at *r* > .95.

**Table 1:** Observed correlations between empirical subscores.

Correlation Pair		2007 Grade 4	2011 Grade 4	2007 Grade 8	2011 Grade 8
θ_1_	θ_2_	.31 (.34)	.45 (.45)	.60 (.56)	.63 (.52)
θ_1_	θ_3_	.58 (.61)	.48 (.42)		
θ_2_	θ_3_	.20 (36)	.15 (.02)		

*Note*: At Grade 4, θ_1_ = General Factor, θ_2_ = Solving for Unknowns, θ_3_ = Fractions and Decimals; at Grade 8, θ_1_ = General Factor, θ_2_ = Algebra and Ratios. Spearman rank-order correlations in parentheses.

To determine whether subscores provide practically distinct information, Feinberg and Wainer [10] suggested the VAR or values-added ratio based on the *PRMSE* of Haberman [11]. Sinharay et al. [12] provided the formula

(1) $$VAR=\frac{r_{1}}{r_{3}^{2}r_{4}},$$

where *r_1_* is subscore reliability, *r_3_* is the disattenuated correlation of the subscore and total score, and *r_4_* is total score reliability. To apply this formula to TIMSS empirical country-level subscores, several equivalences must be assumed. First, from Table 1 of Camilli and Dossey [3], *r_4_* = .96–.99 can be obtained from the first row, assuming the first factor is synonymous with the overall score. As noted above, subscore reliabilities can be obtained as *r_1_* = .89–.95. Finally, disattenuated subscore-total correlations can be obtained from rows 2 and 3 of Table 5 of Camilli and Dossey [3]. For illustration, empirical subscore at 2007 Grade 8 can be examined. In this case, *r_1_* = .96, $r_{3}=.77/\sqrt{.96*.97}=.80$, and *r_4_* = .97, so *VAR* = 1.54. Feinberg and Jurich [13] recommend a minimum value of *VAR* = 1.1 for subscores to be reported. The *VAR*s for the TIMSS empirical subscores exceed this criterion, and this conclusion is unlikely to be altered with plausible changes in the equivalence assumptions above. The *VAR*s were higher at Grade 4 than Grade 8.

To determine whether subscores provide practically information above and beyond the total scale score, Feinberg and Jurich [10] suggested values-added index based on the work of Haberman [11]. We computed these value-added indices for each empirical subscore, and found they all exceeded the threshold recommended by Feinberg and Jurich [13]. The value-added indices were higher at Grade 4 than Grade 8. The reliability results above demonstrate that the empirical subscores are reliable enough to provide diagnostic information, while the value-added analysis indicates the subscores are distinct enough to provide non-overlapping diagnostic information. In short, the empirical subscores were found to be both distinct and informative based on a number of psychometric criteria.
